# Fluorescence-Polarization-Based Assaying of Lysozyme with Chitooligosaccharide Tracers

**DOI:** 10.3390/biom14020170

**Published:** 2024-01-31

**Authors:** Liliya I. Mukhametova, Dmitry O. Zherdev, Anton N. Kuznetsov, Olga N. Yudina, Yury E. Tsvetkov, Sergei A. Eremin, Vadim B. Krylov, Nikolay E. Nifantiev

**Affiliations:** 1 Faculty of Chemistry, M.V. Lomonosov Moscow State University, Leninsky Gory 1/3, 119991 Moscow, Russia; liliya106@mail.ru (L.I.M.); eremin_sergei@hotmail.com (S.A.E.); 2 Laboratory of Glycoconjugate Chemistry, N.D. Zelinsky Institute of Organic Chemistry, Russian Academy of Sciences, Leninsky Prospect 47, 119991 Moscow, Russiatsvetkov@ioc.ac.ru (Y.E.T.); 3 Laboratory of Synthetic Glycovaccines, N.D. Zelinsky Institute of Organic Chemistry, Russian Academy of Sciences, Leninsky Prospect 47, 119991 Moscow, Russia; antonqzn@gmail.com

**Keywords:** fluorescence polarization, peptidoglycan, chitin, lysozyme, chitinase activity

## Abstract

Lysozyme is a well-known enzyme found in many biological fluids which plays an important role in the antibacterial protection of humans and animals. Lysozyme assays are used for the diagnosis of a number of diseases and utilized in immunohistochemistry, genetic and cellular engineering studies. The assaying methods are divided into two categories measuring either the concentration of lysozyme as a protein or its activity as an enzyme. While the first category of methods traditionally uses an enzyme-linked immunosorbent assay (ELISA), the methods for the determination of the enzymatic activity of lysozyme use either live bacteria, which is rather inconvenient, or natural peptidoglycans of high heterogeneity and variability, which leads to the low reproducibility of the assay results. In this work, we propose the use of a chemically synthesized substrate of a strictly defined structure to measure in a single experiment both the concentration of lysozyme as a protein and its enzymatic activity by means of the fluorescence polarization (FP) method. Chito-oligosaccharides of different chain lengths were fluorescently labeled and tested leading to the selection of the pentasaccharide as the optimal size tracer and the further optimization of the assay conditions for the accurate (detection limit 0.3 μM) and rapid (<30 min) determination of human lysozyme. The proposed protocol was applied to assay human lysozyme in tear samples and resulted in good correlation with the reference assay. The use of synthetic fluorescently labeled tracer, in contrast to natural peptidoglycan, in FP analysis allows for the development of a reproducible method for the determination of lysozyme activity.

## 1. Introduction

**Figure 1:**
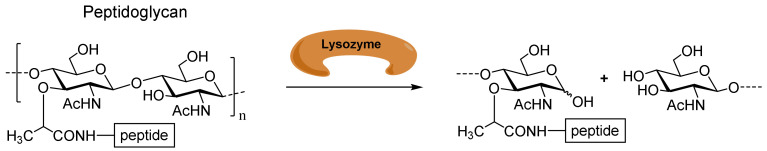
Enzymatic action of lysozyme: catalysis of hydrolysis of the (1→4)-glycosidic bond between *N*-acetyl-muramic acid and *N*-acetyl-glucosamine units in the peptidoglycan.

Lysozyme is an enzyme found in many biological fluids and tissues of animals, such as blood, urine, tears, seminal fluid and milk, and is even produced by some species of fungi and plants. It is often used as a model protein for structural, physicochemical, crystallographic, enzymatic and immunological studies. Lysozyme is known for its antibacterial properties. It hydrolyzes β-(1→4)-glucoside bonds between *N*-acetylmuramic acid and *N*-acetyl-D-glucosamine residues present in the cell wall peptidoglycan of Gram-positive and Gram-negative microorganisms (Figure 1). The active center of lysozyme consists of six binding subcenters for interaction with six monosaccharide units. The Asp52 and Glu35 residues are involved in the catalytic cleavage of glycosidic bonds. Despite the very long history of studying this enzyme, a number of recent publications have discovered new mechanistic features of lysozyme action [1,2,3,4].

The muramidase activity of lysozyme has been supposed to play a key role in its antibacterial properties; however, there is much evidence of the importance of the non-enzymatic mechanisms of lysozyme’s biological actions [5,6]. The lectin-like interaction of lysozyme with the lipopolysaccharide (LPS) O-chain of *Klebsiella pneumonia* has been reported to facilitate its immune defense function [7]. Moreover, lysozyme exhibits antiviral, antitumor and immunomodulatory effects and interacts with antibodies and T-cell receptors [8,9].

The methods developed for the assaying of lysozyme can be divided into two main categories: (1) the determination of lysozyme concentration as a protein and (2) the measurement of its enzymatic activity. The first approach is performed using electrophoretic, chromatographic, immunoenzymatic and spectrophotofluorimetric methods. For example, the enzyme-linked immunosorbent assay (ELISA) has high sensitivity, high specificity and convenience, especially for analyzing large numbers of samples [10]. A resonance light scattering method using CdTe nanoparticles with high sensitivity at the nanogram level is known [11]. Another approach traditionally utilizes live *Micrococcus lysodeikticus* bacteria [12,13]. Lysozyme, by diffusion into agar, catalyzes the hydrolysis of *M. lysodeikticus,* resulting in the formation of a visible clear ring. This method is simple but not very accurate and sometimes requires more than 10 h to complete. The enzymatic activity can also be measured turbidimetrically, nephelometrically or fluorometrically in the liquid phase. The turbidimetric method is simple and fast, but has low sensitivity and a narrow linear range. The determination of lysozyme activity with the resonance light scattering spectral assay has demonstrated high sensitivity and good selectivity [14]. The catalytic activity of lysozyme depends on the pH, ionic strength of the solution, substrate concentration and the content of sodium and potassium in the reaction mixture. All these factors need to be considered when assaying enzymatic activity. Currently, various sensitive test systems have been developed for the determination of lysozyme: a DNA lysozyme aptasensor based on carbon nanotube-modified screen-printed electrodes [15], a graphene oxide- and salicylicylphenylacetylene fluorophore-based sensor [16], and an aptamer immobilized-carbon-nanofiber-modified screen-printed electrode [17]. Another method, label-free lysozyme detection was developed based on surface-enhanced Raman scattering with the molecular imprinting technique (MIP) [18].

The fluorescence polarization (FP) technique is extensively applied to monitor the binding interactions of biomolecules in solution. FP measures protein binding based on the change in the polarization of emitted light when a fluorescently labeled ligand (tracer) is excited by plane-polarized light. When a protein binds to a tracer, there is a change in the polarization of the emitted light due to the slowing of the molecule rotation. The effectiveness of the method depends on the size of the interacting substances; it usually works well if the size of the labeled ligand is much smaller than the size of the interacting protein [19]. The fluorescence polarization (FP) method is used to measure the dissociation constant between receptors and ligands [20] and to study protein-to-carbohydrate [21,22] and protein-to-protein interactions [23]. The usefulness of FP analysis has been demonstrated in high-throughput screening (HTS) for the detection of low-molecular inhibitors of the activity of various enzymes, including kinase [24], phosphatase [25], procaspase [26], endonuclease [27], deacetylase [28] and tissue transglutaminase [29]. FP analysis is being applied to drug discovery, for example, in the identification of new selective inhibitors of the active center of caspases [30], or the development of small molecule inhibitors of the domain reader of DNA methylation [31]. FP has been used also to detect the enzymatic degradation of high-molecular-weight compounds. These tests usually measure enzyme activity levels [32,33,34,35]. The FP method was also applied to determine lysozyme activity using a fluorescein-labeled peptidoglycan of complex structure as a substrate [36]. Subsequently, this method was further used to determine the activity of lysozyme in the synovial fluid of patients with rheumatoid arthritis and osteoarthritis [37].

Various methods for analyzing lysozyme as a protein have been described and investigated. Lysozyme assays serve a range of practical purposes across various fields. In the food industry, lysozyme activity testing is performed for quality control because it is used as a natural preservative in certain foods [38]. Muramidases are also widely used as agricultural feed additives for disease-resistant crops or livestock, which also requires appropriate testing systems [39,40]. The determination of the lysozyme concentration in physiological fluids can be used for the diagnosis of a number of diseases: pleural tuberculosis, intra-abdominal abscesses, lysozyme-associated nephropathy and various conjunctival diseases [41,42,43]. In addition, lysozyme is widely used in medicine [44] and immunohistochemistry, and is utilized in genetic and cellular engineering [9]. However, methods for analyzing lysozyme suffer from disadvantages because the action of lysozyme as a therapeutic agent depends mainly on its activity as an enzyme. On the other hand, the methods described above for the determination of enzymatic activity utilize either live bacteria, which is rather inconvenient, or natural peptidoglycan of high heterogeneity and variability, which makes it difficult to standardize the substrate and leads to the low reproducibility of the assay.

The goal of the described work was to substitute inconvenient peptidoglycan substrates in widely used lysozyme assays and develop a convenient method for lysozyme assaying by fluorescence polarization with the use of synthetic substrates of a strictly defined structure. They included newly synthesized chitin-related tri-, penta- and heptasaccharide (tracers) labelled with fluorescent tags (see Table 1). Synthetic substrates, unlike macromolecular peptidoglycan substrates, have a distinct structure that allow for the performance of enzymatic studies under standardized and reproducible conditions. The advantage of synthetic substrates for the study of glycosidases and glycosyltransferases has been shown in a number of previous studies [45,46,47,48,49].

## 2. Materials and Methods

### 2.1. Reagents and Equipment

Synthetic chitotriose, chitopentaose and chitoheptaose were obtained by us previously [50]. The following commercially available reagents were used: human lysozyme (Sigma-Aldrich, Darmstadt, Germany), fluorescein isothiocyanate (Sigma-Aldrich, Darmstadt, Germany), sodium carbonate (puriss., Chimmed, Moscow, Russia), acetic acid (glacial, puriss., Avilon-Companychim, Moscow, Russia), dimethylformamide (99.8%, Sigma-Aldrich, Darmstadt, Germany) phosphate-buffered saline tablets (Sigma, Darmstadt, Germany). The following proteins and enzymes were used to investigate the specificity of the assay: human urokinase (uPA, Green Cross, Yongin, Republic of Korea), 54 kDa; tissue plasminogen activator (tPA, Dr. Karl Thomae, Biberach an der Riss, Germany); plasmin from human plasma (P1867, Sigma, Darmstadt, Germany); human recombinant lactoferrin, expressed in rice (L1294, Sigma, Darmstadt, Germany); human transferrine, recombinant, expressed in rice (T3705, Sigma, Darmstadt, Germany); trypsin from bovine pancreas (T9935, Sigma, Darmstadt, Germany); human chymotrypsine (SRP6509, Sigma, Darmstadt, Germany). Isolation of conjugates was carried out using reversed-phase Sep-Pak C18 cartridge. Deionized water was prepared with the use of a Simplicity Millipore water purification system (Merck, Burlington, MA, USA). Phosphate buffer (0.01 M sodium phosphate, 0.0027 M KCl, 0.137 M NaCl, pH 7.4) was prepared by dissolution of one saline tablet in 200 mL of distilled water. High-resolution mass spectra were obtained using a Bruker micrOTOF II spectrometer with electrospray ionization (ESI). Both positive ion (capillary voltage of –4500 V) and negative ion spectra (capillary voltage of 3200 V) were recorded. The used mass range (*m*/*z*) was 50–3000 Da; external and internal calibrations were applied (Electrospray Calibrant Solution, Fluka, Buchs, Switzerland). Acetonitrile, methanol or water solutions of analytes were injected through a syringe injector at a flow rate of 3 μL min^–1^. Nitrogen was used as a nebulizer gas (4 L min^–1^); interface temperature was 180 °C. Fluorescence polarization measurements were carried out using the Sentry 200 portable fluorescence polarization reader (Ellie LLC, Germantown, WI, USA) in tube from borosilicate glass, size 10 × 75 mm. Light source was LED, detector was photomultiplier tube, λ_ex_ = 485 and λ_em_ = 535 nm.

### 2.2. Enzymatic Reaction of Oligosaccharide **2a** with Lysozyme

A solution of lysozyme in PBS (300 μL, 5 mg per mL) was added to a solution of pentasaccharide **2a** in PBS (300 μL, 5 mg per mL). After being incubated at 37 °C overnight, the mixture of oligosaccharides and protein was separated by size-exclusion chromatography on TSK HW-40(S) gel using 0.1 M aq. AcOH as eluent. The oligosaccharide fraction was evaporated and subjected to mass spectrometric analysis.

### 2.3. Synthesis of Fluorescein-Labeled Chitooligosaccharides (Tracers)

#### 2.3.1. Synthesis of Trisaccharide Tracer **1b** (Typical Procedure)

To a solution of aminoethyl glycoside **1a** (1.88 mg, 2.53 μmol) and Na_2_CO_3_ (0.80 mg, 7.59 μmol) in distilled water (300 μL), a DMF solution (100 μL) of fluorescein isothiocyanate (FITC) (1.18 mg, 3.04 μmol) was added. The obtained mixture was vigorously mixed and kept at 60 °C for 2 h. The reaction mixture was concentrated *in vacuo*, dissolved in water (300 μL) and the mixture was loaded onto a Sep-Pak C-18 cartridge, which was preliminarily washed with methanol and then with excess water. The cartridge was washed with 2 mL portions of solutions of 0–60 vol.% methanol in water, with the methanol concentration being changed in increments of 5 vol.%. The product was collected in the range of eluent concentrations of 20–40 vol.%, the eluate was concentrated with a rotary evaporator, and the residue was dissolved in water and then lyophilized to give a light orange product (2.61 mg, 96%). High-resolution mass spectrometry (HRMS ESI) calcd. for C_47_H_57_N_5_O_21_S [M+Na]^+^ 1082.3164 found 1082.3159.

#### 2.3.2. Synthesis of Pentasaccharide Tracer **2b**

The fluorescent labelling of aminoethyl glycoside **2a** (1.39 mg, 1.21 μmol) as described for the preparation of trisaccharide **1b**, gave a light orange product (1.57 mg, 83%). HRMS (ESI) calcd. for C_63_H_83_N_7_O_31_S [M+H+Na]^2+^ 744.7412 was found to be 744.7410.

#### 2.3.3. Synthesis of Heptasaccharide Tracer **3b**

To a solution of aminoethyl glycoside **3a** (0.648 mg, 0.437 μmol) and Na_2_CO_3_ (0.35 mg, 3.28 μmol) in a mixture of dimethyl sulfoxide (150 μL) and distilled water (150 μL), a DMSO solution (100 μL) of fluorescein isothiocyanate (0.221 mg, 0.568 μmol) was added. The obtained mixture was vigorously mixed and kept at 60 °C for 2 h. The reaction mixture was concentrated in vacuo, dissolved in water (300 μL) and the mixture was loaded onto a Sep-Pak C-18 cartridge, which was preliminarily washed with methanol and then with excess water. The cartridge was washed with 2 mL portions of solutions of 0–60 vol.% methanol in water, with the methanol concentration being changed in increments of 5 vol.%. The product was collected in the range of eluent concentrations of 10–25 vol.%, the eluate was concentrated with a rotary evaporator, and the residue was dissolved in water and then lyophilized to give a light orange product (0.140 mg, 17%). HRMS (ESI) calcd. for C_79_H_109_N_9_O_41_S [M+Na]^+^ 1894.6339, was found to be 1894.6334.

### 2.4. Fluorescence Polarization Assay

Tracer working solutions from fluorescein-labeled chitooligosaccharides in 10 mM phosphate buffer containing 0.15 M NaCl pH 7.4 were prepared so that the intensity of the solutions exceeded the background signal by 10 times and constituted about 200,000 U. Herein, the tracer concentration was 2.5 nM. Then, 50 µL of lysozyme was added to 950 µL of the tracer working solution to obtain final concentrations of 0.1–10 μM, the reaction mixture was stirred, and the change in the fluorescence polarization signal over time was measured every 30 s. All experiments were duplicated.

### 2.5. Plotting the Calibration Curves

The solutions of recombinant human lysozyme (Sigma) in PBS were used for plotting the calibration curve. The concentration in the stock solution was determined photometrically (the molar extinction coefficient (ε) for lysozyme is 38.940 cm^−1^M^−1^), which was then diluted to obtain a series of standard solutions with a defined concentration. On each curve of the dependence of fluorescence polarization on time, a linear section was selected and the change in the fluorescence polarization signal per unit time (ΔmP/min) was calculated and the maximal value of FP signal (mP_max_) was measured. Then, the dependence of ΔmP/min or mP_max_ on the concentration of lysozyme was plotted. The limit of detection (LOD) was calculated from the regression line as LOD = 3*s*/*b*, where *s*—standard error and *b*—slope of the calibration line.

### 2.6. Lysozyme Quantification in Human Tears

Human lysozyme was determined in tears obtained from volunteers from our laboratory. This study was approved by the Ethics Committee of M.V. Lomonosov Moscow State University. The participants provided their written informed consent to participate in the study. A volume of 50 µL of undiluted tears was added to 950 µL of the tracer working solution and mixed. The mPmax (maximal increasing of FP signal) was fixed and then the change in the FP was measured every 30 s during a period of 4–8 min. All experiments were duplicated. To validate the results of FPIA, all human tear samples were analyzed using the Human Lysozyme ELISA Kit (AssayMax™, Assaypro LLC St. Charles, MO, USA) in accordance with the manufacturer’s instructions.

## 3. Results

The application of the FP method for the qualitative analysis of the enzymatic activity of lysozyme was tested using a series of synthetic fluorescent tracers **1b**–**3b** obtained by the labeling of the aminoethyl oligosaccharides **1a**–**3a** (Table 1). The standard fluorescence labelling protocol in the DMF–water solvent system allows for the preparation of the tri-(**1b**) and pentasaccharides (**2b**) at good yields of 96% and 83%, respectively (entry 1–2). However, in the case of the heptasaccharide **3a**, we failed to obtain labeled product **3b** using the standard procedure due to the low solubility of high-molecular-weight chito-oligosaccharides (entry 3). To overcome this limitation, aqueous DMSO (1:1 *v*/*v*) was used as a reaction solvent, where compound **3a** was moderately soluble. Nevertheless, it gave product **3b** only at a 17% yield (entry 4). This led to the limited applicability of tracer **3b** in analytics, thus making the conjugates of tri-(**1b**) and pentaoside (**2b**) more preferable.

**Figure 2:**
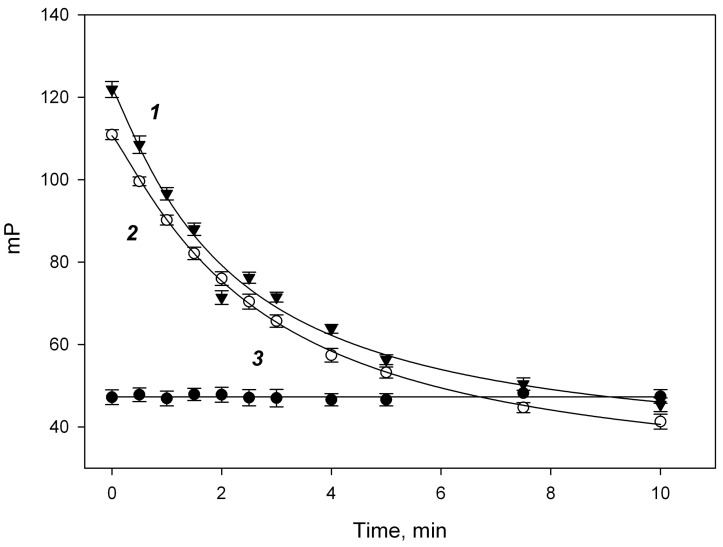
Change in the fluorescence polarization signal (mP) monitored over 10 min after addition of lysozyme into solutions of (1) fluorescein conjugates of heptasaccharide **3b** (initial mP—42.8 ± 1.0), (2) pentasaccharide **2b** (initial mP—40.5 ± 0.8) and (3) trisaccharide **1b** (initial mP—34.2 ± 1.2). The fluorescence intensity was constant during the measurement.

The solutions of fluorescein (FITC) conjugates **1b**–**3b** were prepared so that their fluorescent intensity was 200,000 units, which corresponded to a 10-fold excess of the background signal. The initial concentration of each fluorescently labeled conjugate in the cuvette was 2.5 nM. The initial polarization (mP_0_) for the three FITC conjugates **1b**–**3b** was 34.2 ± 1.2, 40.5 ± 0.8 and 42.8 ± 1.0, respectively. This increase in the FP for the conjugates is likely due to the increase in the molecular weight and size of the fluorescein-labeled chitooligosaccharides. Then, 50 μL of the lysozyme solution was added to these solutions to final concentrations of 0.1–10 μM, and the change in the fluorescence polarization signal was monitored over time (Figure 2). No change in the FP signal was observed for the trisaccharide conjugate **1b**, indicating that its binding to lysozyme did not occur. However, for the penta-**2b** and heptasaccharide **3b**, the FP signal sharply increased almost immediately after mixing the reagents, thus indicating their binding to lysozyme, and this increase was higher for conjugate **3b** than for **2b**. Over time, the FP value decreased, which is probably due to the cleavage of tracer fragments under the action of lysozyme. The specificity of this interaction was studied in the interaction of **2b** with other proteins: lactoferrin and transferrin (concentration up to 1 μM) and enzymes: trypsin, chymotrypsin, plasmin (concentration up to 1 μM), urokinase, tissue plasminogen activator (concentration up to 0.1 μM). However, no change in the FP signal was observed after the mixing of tracer **2b** and the abovementioned proteins and enzymes. Moreover, their presence at the concentration specified did not affect the interaction between tracer **2b** and lysozyme. It evidenced that the FITC-labeled chito-oligosaccharides are specifically cleaved only by lysozyme.

**Figure 3:**
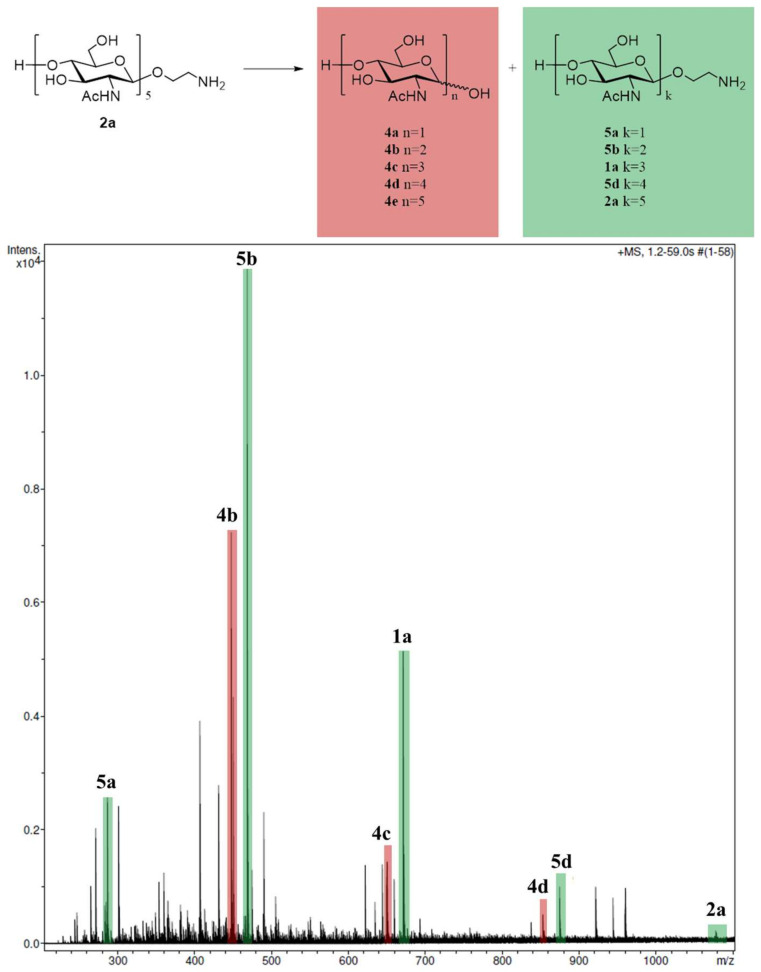
High-resolution mass spectrum of the oligosaccharide mixture obtained after incubation of lysozyme with pentasaccharide **2a**.

To test the chitinase activity of human lysozyme, synthetic pentasaccharide **2a** was incubated with the enzyme at 37 °C for 24 h. After incubation and size exclusion chromatography, the crude mixture of oligosaccharides was analyzed by high-resolution mass spectrometry (HRMS, Figure 3). Peaks corresponding to the oligosaccharide fragments with different sizes of the carbohydrate chain were observed in the mass spectrum, thus proving the ability of lysozyme to hydrolyze the chito-oligosaccharide substrate (Table 2).

**Figure 4:**
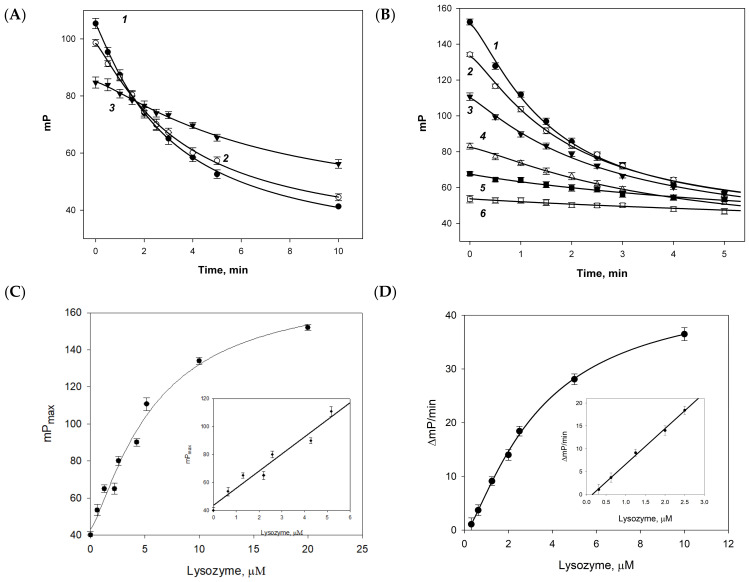
(**A**) Change in the fluorescence polarization signal (mP) monitored over 10 min after addition of lysozyme into solutions of fluorescein conjugate of pentasaccharide **2b** (initial mP—40.5 ± 0.8) at different pH: 6.9 (1), 7.4 (2), and 8.0 (3). (**B**) Change in the fluorescence polarization (mP) signal of fluorescein conjugate of pentasaccharide **2b** (initial mP—40.5 ± 0.8) monitored over 10 min after addition of different amounts of lysozyme: final concentrations 5 (1), 2.5 (2), 1.8 (3), 1.25 (4), 0.625 (5) and 0.312 (6) μM. (**C**) Dependence of the maximal value of the FP signal (mP_max_) measured after addition of lysozyme versus lysozyme concentration. The inset shows the linear range of this curve. (**D**) Dependence of FP signal decrease rate versus lysozyme concentration. The inset shows the linear range of this curve.

Since trisaccharide **1b** did not react with lysozyme, and heptasaccharide **3b** is difficult to obtain and is hardly soluble, the pentasaccharide–FITC conjugate **2b** was chosen for further study. The time dependence of the FP signal for tracer **2b** was studied at pH 6.9, 7.4 and 8.0 (Figure 4A). At pH 6.9 and 7.4, the activity of lysozyme was almost the same. When the pH shifts to a more acidic range, the fluorescence intensity decreases significantly, impeding the measurement of the FP. The pH increase to 8.0 and above causes the decrease of the hydrolysis reaction rate of tracer **2b** by the enzyme. Thus, pH 7.4 was chosen as an optimum for further studies.

The change in the FP signal was studied in the presence of various concentrations of lysozyme from 0.1 to 10 μM (Figure 4B). Thus, the FITC-labeled chitooligosaccharide **2b** was found to initially bind to the enzyme, as evidenced by an increase in the FP signal to the maximal value (mP_max_) that correlates with the lysozyme concentration (Figure 4C). During further incubation, the FP value decreased. It is important to note that the fluorescence intensity remains constant throughout the reaction time.

From the initial linear plots of the FP signal versus time (Figure 4B), the FP signal decrease rate (ΔmP/min, absolute value) was calculated and the dose dependence of the rate of hydrolysis of the fluorescein-labeled chitooligosaccharide **2b** was plotted at various concentrations of the enzyme (Figure 4D). At low enzyme concentrations (0.3–2.5 μM), a linear dose–response correlation was observed, which can be used to determine the lysozyme concentration.

**Figure 5:**
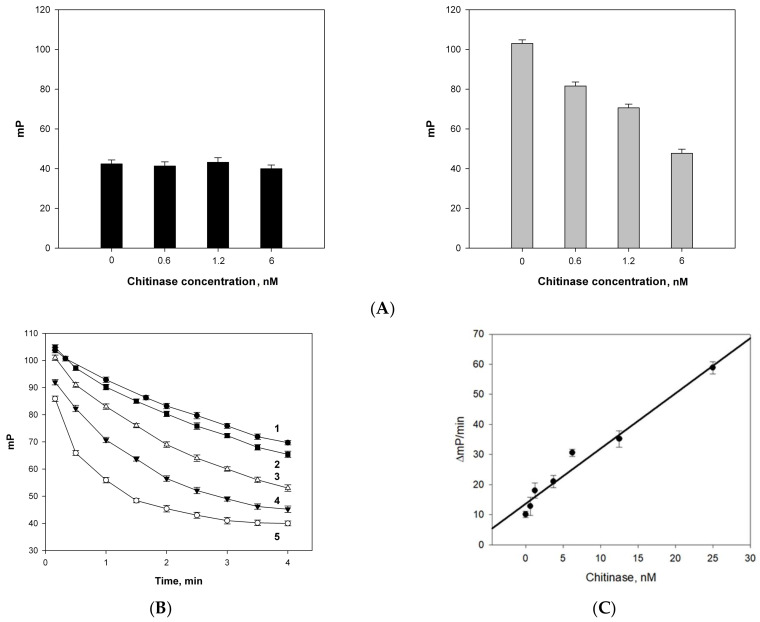
(**A**) No changes in the fluorescence polarization signal (mP) of fluorescein conjugate of pentasaccharide **2b** (initial mP—40.5 ± 0.8) were observed during 10 min after addition of different amounts of chitinase (the FP maximal values (mP_max_) shown on left graph) and observed changes after subsequent addition of fixed amount of lysozyme (mP_max_ shown on right graph). (**B**) Changes in the FP signal of substrate **2b** upon simultaneous action of lysozyme and chitinase: lysozyme concentration is fixed at 8 μM and chitinase concentration changes: 0 (1), 0.625 (2), 1.25 (3), 12.5 (4) and 25 (5) nM. (**C**) The dependence of FP signal decrease rate versus chitinase concentration.

We investigated the applicability of the above method for the determination of the enzymatic activity of commercially available chitinase from *Streptomyces griseus*. Different amounts of chitinase were added to the standard solutions of **2b**, and the FP was measured for 10 min. The fluorescence polarization in this experiment was equal to the value of the initial solution of tracer **2b** (mP~40), and did not change over time and was independent of the amount of chitinase (left plot on Figure 5A). However, after the addition of the standard amounts of lysozyme into each vial, we observed an increase in the FP signal and its maximum value (mP_max_) depended on the amount of chitinase added in the first step (right plot on Figure 5 A). At the lower chitinase concentration, the higher FP signal was detected when the lysozyme was added. The time dependence of the FP signal was measured for the samples, containing the same amounts of tracer **2b** and lysozyme but a variable concentration of chitinase (Figure 5B). The FP signal decrease rates (ΔmP/min, absolute value) were calculated from the kinetic curves on Figure 5B and the dose dependence of the rate versus the chitinase concentration was obtained (Figure 5C). This method allowed for the detection of chitinase at concentrations as low as 1 nM.

The methods for assaying lysozyme based on its ability to bind and to cleave tracer **2b** were used to examine tears obtained from volunteers in our laboratory. After the addition of tear samples into the tracer working solution, the maximal FP signal increase (mP_max_) was fixed and then the change in the FP was measured every 30 s during a period of 4–8 min to obtain the FP signal decrease rate (ΔmP/min). The measured values were used to calculate the amount of lysozyme based on its binding activity (calibration plots on Figure 4C) and enzymatic activity (the calibration plots on Figure 4D). The measured values were used to calculate concentrations based on the calibration plots on Figure 4C,D, respectively. To validate the results of the FP, all the human tear samples were analyzed with ELISA using the Human Lysozyme ELISA Kit (AssayMax™, Assaypro LLC, St. Charles, MO, USA). The obtained results are presented in Table 3.

## 4. Discussion

An effective general approach to determining enzymatic activity can be based on the use of synthetic substrates of a strictly defined structure. Human lysozyme is a type of muramidase (EC 3.2.1.17), the natural substrate of which is a peptidoglycan. Its structure is too complex and heterogeneous for the design of a synthetic muramidase substrate of comparable size with peptidoglycan. However, this enzyme has some side chitinase (EC 3.2.1.14) activity, which arises from the structural similarity between certain fragments of peptidoglycan and chitin. Indeed, the carbohydrate moiety of the peptidoglycan is constructed from β-(1→4)-linked *N*-acetyl-D-glucosamine residues, which is the same for chitin. Natural chitin is insoluble and cannot serve as a substrate for lysozyme, but soluble low-molecular-weight chito-oligosaccharides, structurally related to chitin fragments, can be considered [51] as substrates for assaying lysozyme activity. The effect of lysozyme on chitin oligosaccharides was first described in the 1960s [52] and further kinetic [53] and structural studies [54] have revealed the similarity of the hydrolysis mechanism for chitooligosaccharides and peptidoglycan.

**Figure 6:**
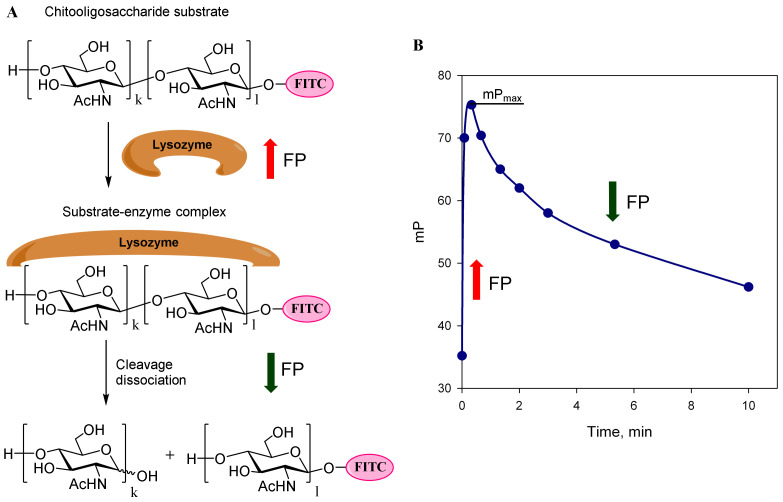
(**A**) The scheme of interaction between lysozyme and chitooligosaccharides. (**B**) Typical view of the time dependence of FP after addition of lysozyme to the chitooligosaccharide tracer **2b**.

The ability of lysozyme to interact with chitooligosaccharides was proven by us with the use of HRMS and PF techniques. The MS analysis showed the possibility of the cleavage of various bonds within the pentasaccharide **2a** (Figure 3). The FP signal rapidly increased to maximal values (mP_max_) after mixing lysozyme and the chito-oligosaccharide tracer, indicating the rapid formation of substrate–enzyme complexes. The subsequent phase of the FP decreases evidenced substrate cleavage and complex dissociation (Figure 6).

The oligosaccharide substrate of optimal size should be small enough to be soluble in water and large enough to bind effectively to the six binding sites in the lysozyme structure. Pentasaccharide **2b** was selected for further studies as the optimal tracer because (1) trisaccharide tracer **1b** did not demonstrate any binding to lysozyme, and (2) heptasaccharide **3b** was poorly soluble in water, making its application difficult (Figure 2, Table 1). The optimization of pH for assaying demonstrated a significant decrease in the enzymatic activity at pH > 8.0 on the one hand, and a low fluorescence intensity in the acidic range on the other hand. Thus, pH 7.4 was selected as an optimal value (Figure 4A). The dependence of the FP signal decrease rate versus lysozyme concentration allowed us to develop a new analytical technique for the quantitative determination of the chitinase activity of lysozyme.

Synthetic chito-oligosaccharide **2b** is able to form a long-lived enzyme–substrate complex with lysozyme, which is slowly converted into dissociation products. In contrast, the lifetime of the complex with chitinase is very short, and our experiment with commercially available chitinase from *Streptomyces griseus* predictably did not reveal any changes in FP. However, adding lysozyme into the enzymatic reaction causes the formation of a lysozyme–substrate complex with the remaining substrate, which increases the FP level (Figure 5A). High concentrations of chitinase result in rapid pentasaccharide **2b** consumption and lower FP signal after the addition of lysozyme. The dose-dependent changes in the FP level for the reaction of pentasaccharide **2b** with chitinase in the presence of fixed amounts of lysozyme shown in Figure 5B allowed for the detection of chitinase at a concentration as low as 1 nM. Hence, the presence of chitinase prevents the measurement of lysozyme concentration by our method and, vice versa, the assaying of chitinase cannot be performed in the presence of lysozyme. It should be noted that chitinases and lysozymes have different origins, and their simultaneous determination in biological fluids is rarely required in practice. In any case, there is demand for the determination of lysozyme in systems which are free of chitinases and where the substitution of heterogenic peptidoglycan substrate by the chemically standard substrate **2b** may increase the reproducibility of the assaying.

The proposed method for the assaying of lysozyme using chito-oligosaccharide tracers allows for the simultaneous measurement of two parameters: the maximum values of the FP peak (mP_max_) and the FP signal decrease rate (ΔmP/min). The first parameter corresponds to the maximum concentration of the enzyme–substrate complex, and the second parameter shows the rate of oligosaccharide cleavage by lysozyme. The corresponding equivalent concentrations of the commercially available recombinant human lysozyme were measured in human tear samples using calibration plots on Figure 4C,D based on the measured mP_max_ and ΔmP/min values (Table 3). As a reference, the concentration of lysozyme as a protein was measured in the tear samples using a commercial ELISA kit (Table 3). The results of the reference assay correlated well with the ones obtained using the mP_max_ values although the latter were proportionally lower, which is probably connected with the use of different standards for plotting the calibration curves. This allows us to conclude that assaying based on the ability of lysozyme to bind **2b** is closer to the determination of lysozyme as a protein. On the other hand, the results obtained using the ΔmP/min values, which are closer to enzymatic activity, were significantly different from those obtained in the reference test that can be expected, because many factors may modify the activity of lysozyme in biological fluids.

## 5. Conclusions

In conclusion, fluorescently labeled conjugates of tri-(**1b**), penta-(**2b**) and heptasaccharide (**3b**) were obtained. The advantages of using pentasaccharide (**2b**) were shown and the conditions for studying the activity of human lysozyme using the fluorescence polarization method were optimized. The obtained results show that the activity of lysozyme could be quickly and simply detected by the fluorescence polarization technique. The use of synthetic fluorescently labeled pentasaccharide (**2b**), in contrast to natural peptidoglycan, in the FP analysis allows for the development of a homogeneous, reproducible method for the determination of lysozyme activity. The detection range of lysozyme is a concentration from 0.35 to 2.5 μM with a detection limit of 0.3 μM. Compared with the known detection methods, the FP method proposed in this work demonstrated better performance and can be applied more effectively for lysozyme detection.

## Figures and Tables

**Table 1:** Synthesis of oligosaccharide tracers **1b**–**3b**.

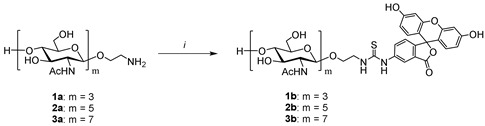				
**Entry**	**Substrate**	**Product**	Conditions * (*i*)	**Yield, %**
1	**1a**	**1b**	FITC (1.2 eq), Na_2_CO_3_ (3 eq), DMF, H_2_O, 60 °C	96
2	**2a**	**2b**	FITC (1.2 eq), Na_2_CO_3_ (3 eq), DMF, H_2_O, 60 °C	83
3	**3a**	**3b**	FITC (1.2 eq), Na_2_CO_3_ (3 eq), DMF, H_2_O, 60 °C	0
4	**3a**	**3b**	FITC (1.3 eq), Na_2_CO_3_ (7.5 eq), DMSO, H_2_O, 60 °C	17

* FITC—fluorescein isothiocyanate; DMF—dimethylformamide; DMSO—Dimethyl sulfoxide.

**Table 2:** Theoretical and found molecular ions *m/z* of the oligosaccharide fragments obtained after cleavage of pentasaccharide **2b** by lysozyme.

Compound	Chemical Formula	Calc.	Found
**4a**	C_8_H_15_NO_6_	244.0792 [M+Na^+^]	not found
**4b**	C_16_H_28_N_2_O_11_	447.1585 [M+Na^+^]	447.1581 [M+Na^+^]
**4c**	C_24_H_41_N_3_O_16_	650.2379 [M+Na^+^]	650.2379 [M+Na^+^]
**4d**	C_32_H_54_N_4_O_21_	853.3173 [M+Na^+^]	853.3172 [M+Na^+^]
**4e**	C_40_H_67_N_5_O_26_	1056.3966 [M+Na^+^]	not found
**5a**	C_10_H_20_N_2_O_6_	287.1214 [M+Na^+^]	287.1291 [M+Na^+^]
**5b**	C_18_H_33_N_3_O_11_	468.2188 [M+H^+^]	468.2184 [M+H^+^]
**1a**	C_26_H_46_N_4_O_16_	671.2982 [M+H^+^]	671.2981 [M+H^+^]
**5d**	C_34_H_59_N_5_O_21_	874.3775 [M+H^+^]	874.3771 [M+H^+^]
**2a**	C_42_H_72_N_6_O_26_	1077.4569 [M+H^+^]	1077.4544 [M+H^+^]

**Table 3:** Determination of human lysozyme in tear samples by various methods.

Sample	ELISA, Commercial Kit, μM	Binding Activity Assay, Based on mP_max_, μM	Enzymatic Activity Assay, Based on ΔmP/min, μM
	(Reference Assay)	(This Study)	
1	79.3 ± 12.4	56.6 ± 8.3	17.2 ± 1.4
2	100.0 ± 14.5	77.9 ± 10.3	24.1 ± 0.7
3	84.8 ± 3.4	64.8 ± 7.6	37.2 ± 1.4

## Data Availability

The data are available from the corresponding author.

## References

[1] Bowman A.L., Grant I.M., Mulholland A.J. (2008). QM/MM Simulations Predict a Covalent Intermediate in the Hen Egg White Lysozyme Reaction with Its Natural Substrate. Chem. Commun..

[2] Hadfield A.T., Harvey D.J., Archer D.B., MacKenzie D.A., Jeenes D.J., Radford S.E., Lowe G., Dobson C.M., Johnson L.N. (1994). Crystal Structure of the Mutant D52S Hen Egg White Lysozyme with an Oligosaccharide Product. J. Mol. Biol..

[3] Held J., van Smaalen S. (2014). The Active Site of Hen Egg-White Lysozyme: Flexibility and Chemical Bonding. Acta Crystallogr. D Biol. Crystallogr..

[4] Vocadlo D.J., Davies G.J., Laine R., Withers S.G. (2001). Catalysis by Hen Egg-White Lysozyme Proceeds via a Covalent Intermediate. Nature.

[5] Levashov P.A., Matolygina D.A., Ovchinnikova E.D., Adamova I.Y., Gasanova D.A., Smirnov S.A., Nelyub V.A., Belogurova N.G., Tishkov V.I., Eremeev N.L. (2019). The Bacteriolytic Activity of Native and Covalently Immobilized Lysozyme against Gram-Positive and Gram-Negative Bacteria Is Differentially Affected by Charged Amino Acids and Glycine. FEBS Open Bio.

[6] Ragland S.A., Criss A.K. (2017). From Bacterial Killing to Immune Modulation: Recent Insights into the Functions of Lysozyme. PLoS Pathog..

[7] Zhang R., Wu L., Eckert T., Burg-Roderfeld M., Rojas-Macias M.A., Lütteke T., Krylov V.B., Argunov D.A., Datta A., Markart P. (2017). Lysozyme’s Lectin-like Characteristics Facilitates Its Immune Defense Function. Q. Rev. Biophys..

[8] Sava G., Benetti A., Ceschia V., Pacor S. (1989). Lysozyme and Cancer: Role of Exogenous Lysozyme as Anticancer Agent. Anticancer Res..

[9] Jiang L., Li Y., Wang L., Guo J., Liu W., Meng G., Zhang L., Li M., Cong L., Sun M. (2021). Recent Insights into the Prognostic and Therapeutic Applications of Lysozymes. Front. Pharmacol..

[10] Vidal M.-L., Gautron J., Nys Y. (2005). Development of an ELISA for Quantifying Lysozyme in Hen Egg White. J. Agric. Food Chem..

[11] Li J., He X.-W., Wu Y.-L., Li W.-Y., Zhang Y.-K. (2007). Determination of Lysozyme at the Nanogram Level by a Resonance Light-Scattering Technique with Functionalized CdTe Nanoparticles. Anal. Sci. Int. J. Jpn. Soc. Anal. Chem..

[12] Berezin I.V., Klyosov A.A., Rabinowitch M.L. (1976). Kinetics of Enzymatic Reactions in Heterogeneous Systems. I. The Kinetic Regularities for the Bacterial Cell *Micrococcus lysodeikticus* Digestion by Lysozyme. Russ. J. Bioorganic Chem..

[13] Hultmark D., Steiner H., Rasmuson T., Boman H.G. (1980). Insect Immunity. Purification and Properties of Three Inducible Bactericidal Proteins from Hemolymph of Immunized Pupae of *Hyalophora cecropia*. Eur. J. Biochem..

[14] Jiang Z.-L., Huang G.-X. (2007). Resonance Scattering Spectra of *Micrococcus lysodeikticus* and Its Application to Assay of Lysozyme Activity. Clin. Chim. Acta Int. J. Clin. Chem..

[15] Rohrbach F., Karadeniz H., Erdem A., Famulok M., Mayer G. (2012). Label-Free Impedimetric Aptasensor for Lysozyme Detection Based on Carbon Nanotube-Modified Screen-Printed Electrodes. Anal. Biochem..

[16] Niamnont N., Jurat N., Sessomboon N., Boonkitpatarakul K., Sukwattanasinitt M. (2018). Salicylicylphenylacetylene Fluorophore Mixed with Graphene Oxide for Selective Lysozyme Detection. J. Lumin..

[17] Senturk H., Eksin E., Işık Ö., İlaslan Z., Mısırlı F., Erdem A. (2021). Impedimetric Aptasensor for Lysozyme Detection Based on Carbon Nanofibres Enriched Screen-Printed Electrodes. Electrochim. Acta.

[18] Ren X., Yang L., Li Y., Cheshari E.C., Li X. (2020). The Integration of Molecular Imprinting and Surface-Enhanced Raman Scattering for Highly Sensitive Detection of Lysozyme Biomarker Aided by Density Functional Theory. Spectrochim. Acta. A Mol. Biomol. Spectrosc..

[19] Hendrickson O.D., Taranova N.A., Zherdev A.V., Dzantiev B.B., Eremin S.A. (2020). Eremin Fluorescence Polarization-Based Bioassays: New Horizons. Sensors.

[20] Prystay L., Gosselin M., Banks P. (2001). Determination of Equilibrium Dissociation Constants in Fluorescence Polarization. J. Biomol. Screen..

[21] Qi J., Oppenheimer M., Sobrado P. (2011). Fluorescence Polarization Binding Assay for *Aspergillus fumigatus* Virulence Factor UDP-Galactopyranose Mutase. Enzyme Res..

[22] Krylov V.B., Petruk M.I., Karimova M.P., Mukhametova L.I., Matveev A.L., Tikunova N.V., Eremin S.A., Nifantiev N.E. (2019). Potential of Fluorescence Polarization Immunoassay for the Detection of *Aspergillus fumigatus* Galactomannan. Russ. Chem. Bull..

[23] James N.G., Jameson D.M. (2014). Steady-State Fluorescence Polarization/Anisotropy for the Study of Protein Interactions. Methods Mol. Biol..

[24] Rohe A., Henze C., Erdmann F., Sippl W., Schmidt M. (2014). A Fluorescence Anisotropy-Based Myt1 Kinase Binding Assay. Assay Drug Dev. Technol..

[25] Pirruccello M., Nandez R., Idevall-Hagren O., Alcazar-Roman A., Abriola L., Berwick S.A., Lucast L., Morel D., De Camilli P. (2014). Identification of Inhibitors of Inositol 5-Phosphatases through Multiple Screening Strategies. ACS Chem. Biol..

[26] Vickers C.J., González-Páez G.E., Umotoy J.C., Cayanan-Garrett C., Brown S.J., Wolan D.W. (2013). Small-Molecule Procaspase Activators Identified Using Fluorescence Polarization. ChemBioChem.

[27] Baughman B.M., Jake Slavish P., DuBois R.M., Boyd V.A., White S.W., Webb T.R. (2012). Identification of Influenza Endonuclease Inhibitors Using a Novel Fluorescence Polarization Assay. ACS Chem. Biol..

[28] Haus P., Korbus M., Schröder M., Meyer-Almes F.-J. (2011). Identification of Selective Class II Histone Deacetylase Inhibitors Using a Novel Dual-Parameter Binding Assay Based on Fluorescence Anisotropy and Lifetime. J. Biomol. Screen..

[29] Hauser C., Wodtke R., Löser R., Pietsch M. (2017). A Fluorescence Anisotropy-Based Assay for Determining the Activity of Tissue Transglutaminase. Amino Acids.

[30] An J., Kim S.Y., Yang E.G., Chung H.S. (2022). A Fluorescence-Polarization-Based Lipopolysaccharide-Caspase-4 Interaction Assay for the Development of Inhibitors. Molecules.

[31] Kalmode H.P., Podsiadly I., Kabra A., Boulton A., Reddy P., Gao Y., Li C., Bushweller J.H. (2022). Small-Molecule Inhibitors of the MLL1 CXXC Domain, an Epigenetic Reader of DNA Methylation. ACS Med. Chem. Lett..

[32] Schade S.Z., Jolley M.E., Sarauer B.J., Simonson L.G. (1996). BODIPY-Alpha-Casein, a pH-Independent Protein Substrate for Protease Assays Using Fluorescence Polarization. Anal. Biochem..

[33] Cupp-Enyard C. (2009). Use of the Protease Fluorescent Detection Kit to Determine Protease Activity. J. Vis. Exp..

[34] Kinoshita K., Maeda H., Hinuma Y. (1980). Fluorescence Polarization Assay of Plasmin, Plasminogen, and Plasminogen Activator. Anal. Biochem..

[35] Glukhikh A.S., Shevelkova A.N., Eremin S.A., Sinitsyn A.P. (1991). Use of the Method of Fluorescence Polarization to Determine the Degree of Order in the Action of Amylases. Biochem. Mosc..

[36] Maeda H. (1980). A New Lysozyme Assay Based on Fluorescence Polarization or Fluorescence Intensity Utilizing a Fluorescent Peptidoglycan Substrate. J. Biochem..

[37] Okamoto T., Ueda K., Maeda H., Kambara T. (1987). Determination of Lysozyme Activity by Fluorescence Polarization in Rheumatoid Synovial Fluids and Release of Lysozyme from Polymorphonuclear Leukocytes by Chemotactic Factors. J. Immunol. Methods.

[38] Nawaz N., Wen S., Wang F., Nawaz S., Raza J., Iftikhar M., Usman M. (2022). Lysozyme and Its Application as Antibacterial Agent in Food Industry. Molecules.

[39] Wang Y., Goossens E., Eeckhaut V., Calvo E.P., Lopez-Ulibarri R., Eising I., Klausen M., Debunne N., De Spiegeleer B., Ducatelle R. (2021). Dietary Muramidase Degrades Bacterial Peptidoglycan to NOD-Activating Muramyl Dipeptides and Reduces Duodenal Inflammation in Broiler Chickens. Br. J. Nutr..

[40] Deng J., Bi B., An Q., Kong L., Wang Q., Tao L., Zhang X. (2012). Effect of Dietary Inclusion of Lysozyme on Growth Performance and Plasma Biochemical Parameters of Rainbow Trout (*Oncorhynchus mykiss*). Aquac. Nutr..

[41] Kudose S., Cossey L.N., Canetta P.A., Sekulic M., Vanbeek C.A., Huls F.B., Gupta I., Bu L., Alexander M.P., Cornell L.D. (2023). Clinicopathologic Spectrum of Lysozyme-Associated Nephropathy. Kidney Int. Rep..

[42] Helal R., Melzig M.F. (2008). Determination of Lysozyme Activity by a Fluorescence Technique in Comparison with the Classical Turbidity Assay. Pharm.-Int. J. Pharm. Sci..

[43] Aggarwal A.N., Agarwal R., Dhooria S., Prasad K.T., Sehgal I.S., Muthu V. (2022). Pleural Fluid Lysozyme as a Diagnostic Biomarker of Pleural Tuberculosis: A Systematic Review and Meta-Analysis. Lung India Off. Organ Indian Chest Soc..

[44] Daniel M.P., Gaikwad V., Verghese M., Abraham R., Kapoor R. (2015). Serum Lysozyme (Muramidase) Levels in Intra-Abdominal Abscesses: An Experimental Study. Indian J. Surg..

[45] Zhong W., Kuntz D.A., Ember B., Singh H., Moremen K.W., Rose D.R., Boons G.-J. (2008). Probing the Substrate Specificity of Golgi Alpha-Mannosidase II by Use of Synthetic Oligosaccharides and a Catalytic Nucleophile Mutant. J. Am. Chem. Soc..

[46] Silchenko A.S., Ustyuzhanina N.E., Kusaykin M.I., Krylov V.B., Shashkov A.S., Dmitrenok A.S., Usoltseva R.V., Zueva A.O., Nifantiev N.E., Zvyagintseva T.N. (2017). Expression and Biochemical Characterization and Substrate Specificity of the Fucoidanase from *Formosa algae*. Glycobiology.

[47] Ayers J.D., Lowary T.L., Morehouse C.B., Besra G.S. (1998). Synthetic Arabinofuranosyl Oligosaccharides as Mycobacterial Arabinosyltransferase Substrates. Bioorg. Med. Chem. Lett..

[48] Sethi M.K., Buettner F.F.R., Krylov V.B., Takeuchi H., Nifantiev N.E., Haltiwanger R.S., Gerardy-Schahn R., Bakker H. (2010). Identification of Glycosyltransferase 8 Family Members as Xylosyltransferases Acting on O-Glucosylated Notch Epidermal Growth Factor Repeats. J. Biol. Chem..

[49] Sethi M.K., Buettner F.F.R., Ashikov A., Krylov V.B., Takeuchi H., Nifantiev N.E., Haltiwanger R.S., Gerardy-Schahn R., Bakker H. (2012). Molecular Cloning of a Xylosyltransferase That Transfers the Second Xylose to O-Glucosylated Epidermal Growth Factor Repeats of Notch. J. Biol. Chem..

[50] Yudina O.N., Tsvetkov Y.E., Nifantiev N.E. (2015). Synthesis of 2-Aminoethyl Glycosides of Chitooligosaccharides. Russ. Chem. Bull..

[51] Song H., Inaka K., Maenaka K., Matsushima M. (1994). Structural Changes of Active Site Cleft and Different Saccharide Binding Modes in Human Lysozyme Co-Crystallized with Hexa-N-Acetyl-Chitohexaose at pH 4.0. J. Mol. Biol..

[52] Powning R.F., Irzykiewicz H. (1966). The Effect of Lysozyme on Chitin Oligosaccharides. Biochim. Biophys. Acta.

[53] Holler E., Rupley J.A., Hess G.P. (1975). Productive and Unproductive Lysozyme-Chitosaccharide Complexes. Kinetic Investigations. Biochemistry.

[54] Roman D.L., Ostafe V., Isvoran A. (2020). Deeper inside the Specificity of Lysozyme When Degrading Chitosan. A Structural Bioinformatics Study. J. Mol. Graph. Model..

